# Impact of Race and Ethnicity on Presentation and Surgical Outcomes of Idiopathic Macular Holes

**DOI:** 10.3390/jpm12091518

**Published:** 2022-09-16

**Authors:** Matthew N. Parvus, Abigail M. Louis, Stephanie Trejo Corona, Tien P. Wong, James C. Major, Sagar B. Patel, Charles C. Wykoff

**Affiliations:** 1 Retina Consultants of Texas, Retina Consultants of America, Houston, TX 77401, USA; 2 John P. and Kathrine G. McGovern Medical School at UT Health Houston, Houston, TX 77030, USA; 3 Blanton Eye Institute, Houston Methodist Hospital, Houston, TX 77030, USA

**Keywords:** idiopathic macular hole, ethnicity, race, diversity, retina

## Abstract

There is limited data on the presentation and surgical outcomes of idiopathic macular holes (IMH) for different ethnic and racial groups. Recognition of distinct, clinically-relevant patient subgroups may provide opportunities to identify specific unmet needs including possible barriers to optimal healthcare delivery. Medical records of patients who underwent surgery for IMH (between 2016 and 2022) at a large, urban retina practice were reviewed and self-reported ethnicity (Hispanic and non-Hispanic) and race (Asian, Black, White, and Other) were captured. The primary variables included (1) mean minimum linear diameter (MLD) at presentation and (2) surgical outcome (IMH closure status). Overall, mean MLD for all study eyes (515) was 366.1 μm, and surgical success was achieved in 489 (95.0%) eyes. Hispanic eyes presented with larger mean MLD (*p* = 0.002) compared to non-Hispanic eyes. Asian, Black, and Other eyes presented with larger mean MLD (*p* = 0.033, *p* < 0.001, *p* < 0.001) when compared to White eyes. The presentation of IMH varied in severity among different ethnic and racial groups. Hispanic patients were found to have worse preoperative visual acuity (VA), longer time to surgery, and larger mean MLD and BD compared to non-Hispanic participants. Black and Other patients were found to have worse VA, time to surgery, and larger mean MLD and BD when compared to White participants.

## 1. Introduction

A full-thickness macular hole (FTMH) is a structural condition characterized by a full-thickness neurosensory defect in the central macula. The majority of cases are idiopathic macular holes (IMH) or presumed to be secondary to vitreomacular traction [1]. Visual impairment varies but tends to increase in severity over time. In most cases, the recommended treatment for FTMH is pars plana vitrectomy (PPV) with internal limiting membrane (ILM) peeling [2]. Decreased time to surgery for FTMH (<2 months) has been associated with greater visual acuity (VA) improvement (3.94 lines vs. 2.96 lines) and anatomic surgical success (100% vs. 85%) than surgery performed later (>6 months) [3]. 

Some studies have demonstrated variation in the presentation and surgical outcomes for different races [4,5,6], with limited data regarding the Hispanic population. Despite being the largest minority group in the United States (62.1 million, 18.7% of the U.S. population in 2020) [7], the Hispanic population remains significantly medically underserved [8,9]. For example, an analysis of the Los Angeles Latino Eye Study (LALES) revealed that a high prevalence (about 63%) of the Latino population had undetected eye disease (97.9% age-related macular degeneration (AMD), 95.0% diabetic retinopathy, 82.4% open-angle glaucoma, and 57.0% cataract) [9]. 

Ethnicity and race are not synonymous [10]. In the setting of health research, ethnicity is considered a social-political construct referring to sharing of common culture, while race consists of personal and group identity as more familiar biological indicators [10]. Recognition of distinct and clinically relevant patient subcategories may allow the identification of specific health needs for different subgroups [8,10,11]. 

Obtaining a better understanding of variable presentations and outcomes of IMH across different populations could identify associations or health disparities contributing to significant vision loss resulting from undetected ocular disease. 

## 2. Materials and Methods

This retrospective comparative cohort study was approved by the Institutional Review Board of Houston Methodist Hospital, Houston, Texas. Medical records were reviewed for patients presenting with an IMH between July 2016 and June 2022 at a large, urban, retina practice (Retina Consultants of Texas, Houston, TX, USA). The following ICD-10 codes for macular cyst, hole, or pseudohole in the right, left, or unspecified eye were used to identify patients: H35.341, H35.342, and H35.349. CPT code 67042 was used to identify patients who underwent repair with PPV. All study procedures adhered to the tenets set forth in the Declaration of Helsinki and the Health Insurance Portability and Accountability Act.

Eyes with prior PPV, prior intravitreal injections, lamellar macular hole as the reason for surgery, or poor preoperative imaging were excluded. Poor imaging included images that were unclear and did not have distinct features necessary for reliable measurement. Patients with myopic degeneration, high myopia, AMD, diabetic retinopathy, and a prior diagnosis of a lamellar hole that progressed to IMH prior to surgical intervention were included. 

The following 13 metrics were recorded from the medical records for each patient: (1) age at the date of surgery, (2) self-reported ethnicity, (3) self-reported race, (4) primary language spoken, (5) first date of FTMH diagnosis, (6) VA at preoperative visit, (7) minimum linear diameter (MLD) of IMH at preoperative visit, (8) basal diameter (BD) of IMH at preoperative visit, (9) lens status in the eye with IMH at preoperative visit, (10) presence of fellow eye IMH at any time, (11) diabetes status at the time of diagnosis, (12) open or closed status of FTMH postoperatively, and (13) postoperative visits and VA (month 1, 6, and 12; each time-point defined as within an 8-week window around the respective postoperative visit). 

**Figure 1:**
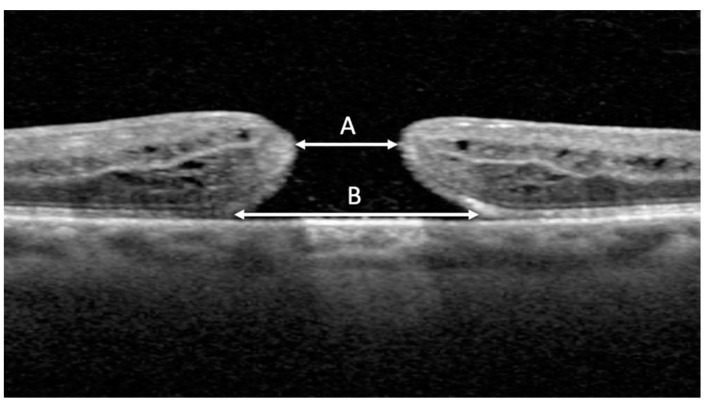
OCT image measurement with distance overlay tool. Minimum linear diameter (MLD) (**A**) was measured between nearest ends of broken macular tissue in the scan with the widest hole dimensions. Basal diameter (BD) (**B**) was measured at the level of the retinal pigment epithelium.

MLD, BD, and closure status following surgery were measured using spectral domain-optical coherence tomography (OCT) imaging (Heidelberg Spectralis, Heidelberg Engineering, Heidelberg, Germany) with a volume-per-cube acquisition protocol (20° × 20°, 49 B-scans, 768 A-scans per line) and 9 times image averaging. MLD was measured in 1:1 μm format using the measure distance overlay tool. MLD was measured between the nearest ends of broken macular tissue in the scan with the widest hole dimensions [12] (Figure 1). Any operculated component of the hole was excluded from the MLD measurement [12]. BD was measured at the level of the retinal pigment epithelium layer between the broken macular tissue in the same scan that the MLD measurements were taken [12]. MLD and BD measurements were conducted by an independent investigator (MNP), and consensus with a second grader (CCW) was performed for cases that were perceived as unclear by the initial investigator. MLD was categorized into three size ranges: <250 μm (small), 250–400 μm (medium), and >400 μm (large) [13] based on traditional macular hole staging [1,12]. BD was also categorized into three size ranges: <577.0 μm (small), 577.0–890.3 μm (medium), and >890.3 μm (large). These groups were created by dividing the range of BD measurements from the study group equally into thirds. 

Race and ethnicity were reported based on two questions on the patient intake questionnaire. Ethnicity was self-reported by the patient as “Hispanic or Latino” (hereafter, Hispanic), “Not Hispanic or Latino” (hereafter, non-Hispanic), or “Declined to Specify.” Race was self-reported by the patient as “Asian,” “Black or African American” (hereafter, Black), “White/Caucasian” (hereafter, White), or “Other Race” (hereafter, Other). Those who declined to specify ethnicity were not included in the study. Preferred language was analyzed, comparing English and non-English speakers as well as Spanish and non-Spanish speakers.

The primary variables measured included MLD and closure status of the IMH following surgery. MLD was measured at the preoperative visit. Surgical success was defined as anatomic closure upon the first attempt. Surgical failure was defined as no anatomic closure of the IMH up to one year postoperatively or IMH that required secondary surgery. IMH closure was evaluated by OCT and chart review at postoperative visits (month 1, 6, and 12). Preoperative VA was recorded at the preoperative visit, and postoperative VA was recorded at months 1, 6, and 12. VA was converted to logarithm of the minimum angle of resolution (logMAR) values for analysis. Time to surgery was also compared and defined as time (in days) from the date of initial presentation of diagnosis to date of surgery. 

Statistical analysis was performed using RStudio software version 4.2.1 (www.rstudio.com (accessed on 20 July 2022), Boston, MA, USA). Linear regression analysis was used to identify associations between IMH presentation and surgical outcome variability between different racial and ethnic groups. Student’s t-test and analysis of variance were used to determine statistically significant differences between means. Chi-squared test was used to compare proportional data. A *p*-value of less than 0.05 was considered statistically significant.

## 3. Results

### 3.1. Baseline Cohort Data

A total of 515 eyes from 488 patients were identified that met the criteria for inclusion. The mean age was 66.3 years (SD = 7.5) and the majority were female (71.8%; *p* < 0.001) (Table 1). For ethnicity, there were 79 (15.3%) Hispanic and 436 (84.7%) non-Hispanic study eyes. There were 56 (70.9%) female and 23 (29.1%) male eyes in the Hispanic group (*p* < 0.001) compared to 314 (72.0%) female and 122 (28.0%) male eyes in the non-Hispanic group (*p* < 0.001). For race, there were 23 (4.5%) Asian, 79 (15.3%) Black, 365 (70.9%) White, and 48 (9.3%) Other study eyes. There were 16 (69.6%) female and 7 (30.4%) male eyes in the Asian group (*p* = 0.061), 62 (78.5%) female and 17 (21.5%) male eyes in the Black group (*p* < 0.001), 255 (69.9%) female and 110 (30.1%) male eyes in the White group (*p* < 0.001), and 37 (77.1%) female and 11 (22.9%) male eyes in the Other group (*p* < 0.001). Thirty-three eyes were from Spanish-speaking participants (6.4%) and 475 eyes were from English-speaking participants (92.2%); 7 (1.4%) eyes were from participants who spoke another language, categorized as “Other Language”. There were three different languages spoken by the participants included in the “Other Language” category: Chinese, Vietnamese, and Japanese. 

Regarding clinical analysis, 164 (31.8%) study eyes were pseudophakic at their preoperative visit. There were 55 (10.7%) eyes with fellow eye (FE) FTMH and 23 (5.5%) eyes with FE lamellar macular hole. There were 2 (0.4%) subjects with type I diabetes (DMI) and 78 (15.1%) patients with type II diabetes (DMII). Statistical analysis compared non-diabetic study eyes to diabetic (DMI and DMII) study eyes. 

### 3.2. Preoperative Status

#### 3.2.1. Time to Surgery

**Figure 2:**
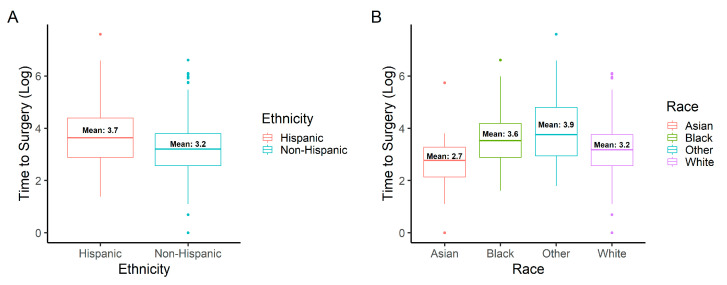
Logarithmic time to surgery distribution by (**A**) ethnicity (Hispanic or non-Hispanic) and (**B**) race (Asian, Black, White, or Other).

Time to surgery was significantly longer for Hispanic (99.7 days) study eyes than non-Hispanic (43.2 days) study eyes (*p* = 0.046). Time to surgery was longer for Black (64.2 days) and Other (135.9 days) study eyes when compared to White (39.5 days) study eyes (*p* = 0.046, *p* = 0.037, respectively). Linear regression analysis demonstrated a positive association between MLD and time to surgery (*p* = 0.032; R^2^ = 0.01) but none between BD and time to surgery (*p* = 0.276; R^2^ < 0.01). A summary of time to surgery findings can be found in Table 2. The distribution of time to surgery between ethnicity and race can be observed in Figure 2.

#### 3.2.2. Preoperative Vision

Regarding preoperative VA (Table 3), the overall logMAR mean VA was 0.86 (Snellen = 20/146). Hispanic study eyes had significantly worse VA (logMAR = 1.00; Snellen = 20/198) compared to non-Hispanic (logMAR = 0.84; Snellen = 20/138) study eyes (*p* = 0.008). VA was significantly different amongst different racial groups (*p* < 0.001). Preoperative VA for Asian (logMAR = 1.01; Snellen = 20/205), Black (logMAR = 0.95; Snellen = 20/179), and Other (logMAR = 1.10; Snellen = 20/249) study eyes were significantly worse when compared to White (logMAR = 0.81; Snellen = 20/128) study eyes (*p* = 0.045, *p*= 0.007, *p* < 0.001, respectively). Preoperative VA was significantly worse for subjects with subsequent unsuccessful surgery (*p* = 0.003).

### 3.3. Postoperative Status

#### 3.3.1. IMH Measurements

Overall, the mean MLD and BD for the cohort was 366.1 μm and 749.0 μm, respectively. Categorizing MLD into ranges, 167 (32.4%) were small, 148 (28.8%) were medium, and 200 (38.8%) were large (*p* = 0.018). An association was identified between time to surgery and MLD (*p* = 0.032; R^2^ = 0.01), but not between time to surgery and BD (*p* = 0.276; R^2^ < 0.01).

**Figure 3:**
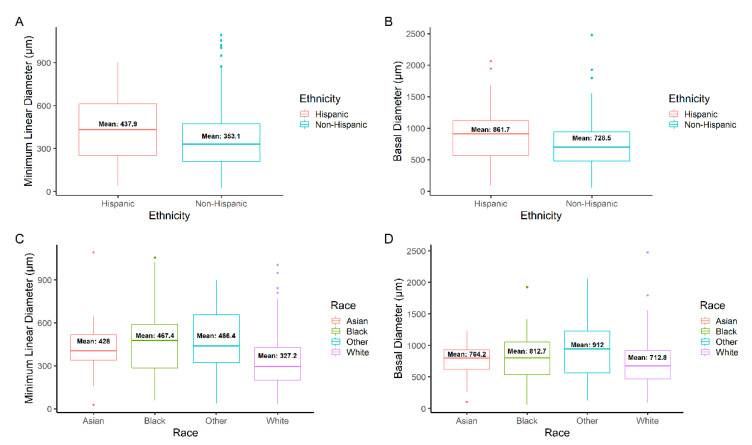
Minimum linear diameter (MLD) distribution by (**A**) ethnicity (Hispanic or non-Hispanic) and (**C**) race (Asian, Black, White, or Other). Basal diameter (BD) distribution by (**B**) ethnicity and (**D**) race. All MLD and BD measurements were taken preoperatively.

MLD was significantly larger for Hispanic (437.9 μm) compared to non-Hispanic (353.2 μm) study eyes (*p* = 0.002) (Table 4, Figure 3A). Regarding race, there was a significant difference in MLD between Asian (428.0 μm), Black (467.4 μm), White (327.2 μm), and Other (466.4 μm) study eyes (*p* < 0.001) (Figure 3C). Study eyes of Asian, Black, and Other had significantly larger mean MLD when compared to White study eyes (*p* = 0.033, *p* < 0.001, *p* < 0.001, respectively). Significant differences in mean MLD amongst study eyes of English and non-English speakers (*p* = 0.007) and study eyes of Spanish and non-Spanish speakers (*p* = 0.007) were found. There was a significant difference in mean MLD for the Hispanic study eyes with (14; 17.7%) and without (65; 82.3%) diabetes (514.2 μm vs. 421.5 μm; *p* = 0.046). BD measurements were recorded and analyzed in addition to MLD. BD was significantly larger for the Hispanic (861.7 μm) compared to the non-Hispanic (728.5 μm) study eyes (*p* = 0.009) (Figure 3B). Regarding race, there was a significant difference in BD between Asian (764.2 μm), Black (812.7 μm), White (712.8 μm), and Other (912.0 μm) study eyes (*p* < 0.001) (Figure 3D). Study eyes of Black and Other presented with significantly larger BD when compared to White study eyes (*p* = 0.023, *p* = 0.005, respectively). Linear regression analysis demonstrated a positive association between MLD and BD (*p* < 0.001; R^2^ = 0.6).

Beyond comparing the mean MLD and BD, the numbers of small, medium, and large measurements for each group were also analyzed. Significantly more Hispanic study eyes had a large MLD (45, 57.0%; *p* < 0.001) than non-Hispanic (155, 35.6%; *p* = 0.462) study eyes (Odds Ratio (OR) 1.60; 95% Confidence Interval (CI), 1.04–2.45; *p* = 0.028) (Table 5). For race, there was a greater number of Black (46, 58.2%; *p* < 0.001) and Other (30, 62.5%; *p* < 0.001) study eyes that had large MLD measurements amongst race categories (Table 6). Regarding BD, significantly more Hispanic study eyes were found to have large BD measurements (41, 52.0%; *p* = 0.002) than non-Hispanic (131, 30.0%; *p* = 0.346) study eyes (OR 1.73; 95% CI 1.10–2.69; *p* = 0.015). Only Other (30, 62.5%; *p* < 0.001) study eyes were found to have significantly larger BD measurements than Asian, Black, or White study eyes.

#### 3.3.2. Surgical Outcomes

Regarding IMH closure, unsuccessful surgery outcomes occurred in 26 (5.0%) study eyes. Of these 26 eyes, 8 (30.8%) had one additional surgery and 1 (3.8%) had two additional surgeries. Of these 9 eyes that had additional surgery, 7 (77.8%) successfully closed. MLD was significantly larger for study eyes with failed (607.3 μm) compared to successful (353.3 μm) surgical outcomes (*p* < 0.001). No significant difference was found between IMH closure rates among study eyes of different ethnic (*p* = 0.174) or racial (*p* = 0.426) groups. The eyes of English speakers did not have significantly more successful surgical outcomes (23; 4.8%) than eyes of non-English (3; 7.5%) speakers (*p* = 0.718). The same was true for eyes of Spanish speakers, as they did not have more successful surgical outcomes (3; 9.1%) than eyes of non-Spanish (23; 4.8%) speakers (*p* = 0.493). While Spanish speakers did not have statistically significant differences in successful surgical outcomes, it is important to note the proportion of failed surgeries was 1.9 times higher than non-Spanish speakers. A summary of outcome measures can be found in Table 7. 

#### 3.3.3. Follow-Up Data

**Figure 4:**
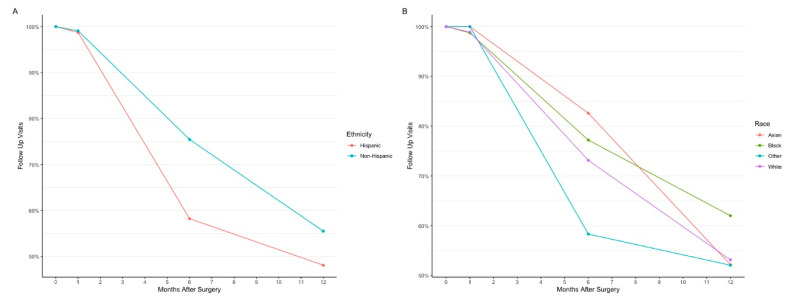
Comparison of follow-up visits by (**A**) ethnicity (Hispanic or non-Hispanic) and (**B**) race (Asian, Black, White, or Other). The four time points indicated are the preoperative visit (month 0), 1-month postoperative visit, 6-month postoperative visit, and 12-month postoperative visit.

During follow-up, data was available for 510 (99.0%) subjects at month 1, 375 (72.8%) at month 6, and 280 (54.4%) at month 12. Amongst the Hispanic population, there is a trend of decreased follow-up (46; 58.2% of Hispanic patients who received surgery), predominantly 6 months postoperatively (Figure 4A), when compared to non-Hispanics (329; 75.5% of non-Hispanic patients who received surgery), although this difference was not statistically significant. This trend was also observed in study eyes of Other at 6 months. For Other, there were 28 (58.3%) follow-up visits for study eyes that received surgery compared to 19 (82.6%) Asian, 61 (77.2%) Black, and 267 (73.2%) White study eyes (Figure 4B). 

#### 3.3.4. Visual Outcomes

**Figure 5:**
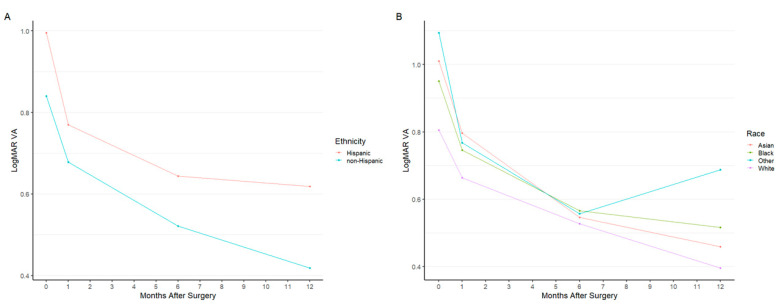
Mean logMAR visual acuity by (**A**) ethnicity (Hispanic or non-Hispanic) and (**B**) race (Asian, Black, White, or Other). The four time points indicated are the preoperative visit (month 0), 1-month postoperative visit, 6-month postoperative visit, and 12-month postoperative visit.

Postoperative VA was recorded at months 1, 6, and 12 (Table 8). Between ethnicities, a significant difference in VA was only observed at the 12-month postoperative visit (*p* = 0.026) (Figure 5A). Similar findings were observed amongst different racial groups, as a significant difference was only observed at the 12-month period (*p* = 0.005) (Figure 5B). Postoperative VA for eyes that failed surgery were significantly worse at all three postoperative time points (*p* < 0.001, *p* < 0.001, *p* < 0.001, respectively).

**Figure 6:**
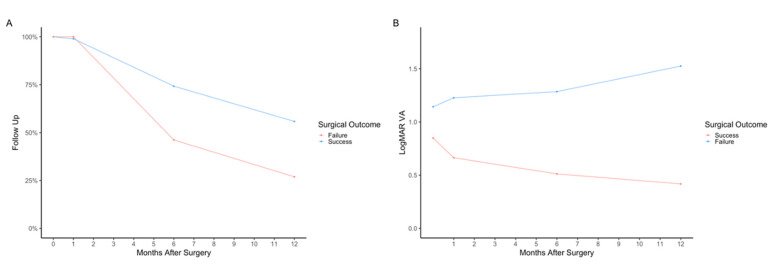
Comparison of successful and unsuccessful surgery outcomes by (**A**) follow-up visits attended (in percentage total) and (**B**) mean logMAR visual acuity. The four time points indicated are the preoperative visit (month 0), 1-month postoperative visit, 6-month postoperative visit, and 12-month postoperative visit.

Of the patients that had successful surgery, attendance rates for follow-up visits at months 1, 6, and 12 were 484 (99.0%), 363 (72.4%), and 273 (55.8%), respectively. For patients with failed surgery, attendance rates for follow-up visits at months 1, 6, and 12 were 26 (100%), 12 (46.2%), and 7 (26.9%), respectively (Figure 6A). Differences in logMAR VA were significantly different at all follow-up visits between those with successful surgery and those with unsuccessful surgery (*p* < 0.001 at month 1, *p* < 0.001 at month 6, and *p* < 0.001 at month 12 follow-up visits) (Figure 6B). When evaluating characteristics between those who kept follow-up visits at 6 months compared to those who stopped follow-up at 6 months, no significant differences were found between MLD, BD, time to surgery, or VA at the 1-month postoperative visit (*p* = 0.093, *p* = 0.194, *p* = 0.371, and *p* = 0.235, respectively). 

## 4. Discussion

This current retrospective analysis of 515 eyes with IMH found clinically meaningful differences in baseline characteristics by ethnic and racial groups. For example, Hispanic patients had greater mean MLD and BD of IMH, worse preoperative VA, and longer time to surgery when compared to non-Hispanic patients. Similarly, significant differences in IMH size and time to surgery were identified when comparing patients of different racial groups. It is possible that both delayed presentation to clinic as well as increased time to surgery are both factors contributing to increased IMH size. 

### 4.1. Association between Variance in MLD and BD, Time to Surgery, and Surgical Outcomes

The IMH size analysis of the Hispanic population in this study suggests that Hispanic study eyes, in general, suffer from a disproportionately large number of large IMH. Delay in ophthalmic care has been proposed to lead to a larger IMH size [3,6,14]. The Hispanic cohort in the current study had a significantly longer time to surgery, which could be the underlying cause for the larger IMH observed on presentation. IMH MLD and BD increase over time, especially for small IMH, which have been reported to increase at a rate of 1.67 μm per day [14]. This delay may be related to well-described disparities in access to care for minority populations [8,9,15,16,17]. A study of 183,054 respondents using the Medical Expenditure Panel Survey from 2007 to 2015 reported that Hispanic (OR 0.72; 95% CI 0.66–0.78) and Black (OR 0.74; 95% CI 0.69–0.79) patients were significantly less likely to visit an outpatient ophthalmologist when compared to non-Hispanic White patients [18]. It is difficult to determine whether this phenomenon would impact time to initial presentation to clinic vs. time from presentation to surgery, but the significantly longer time to surgery observed for eyes of Hispanic and Black patients in this study presents evidence for the latter. Additionally, a trend of decreased follow-up at the 6-month postoperative visit was observed for the Hispanic population. 

Regarding time to surgery for different racial groups, study eyes of Black and Other were found to have increased time to surgery when compared with White study eyes. That being said, this was not true for the Asian study eyes, which had the shortest mean time to surgery of all four racial groups (30.6 days). This highlights the importance of attempting to identify specific needs of different minority groups in order to optimally address disparities.

It has been reported that increased time to surgery is associated with worse surgical outcomes [3]. In addition, the current study demonstrated a positive association between MLD and time to surgery, emphasizing the importance of prompt treatment and management for patients with IMH. A possible reason for longer time to surgery is disparities in rates of healthcare insurance which has been observed in Hispanic and Black populations [19]. According to the LALES study, 30% of Latinos were uninsured and 21% were publicly insured [9]. A lack of healthcare coverage has been associated with worse health outcomes, advanced severity of disease, and increased morbidity and mortality [19].

Although no differences were found between different ethnic and racial groups, larger mean MLD and BD were associated with worse outcomes in the study cohort overall. This is consistent with the previous literature that supports MLD as a prognostic factor for surgical outcomes [20,21,22]. Time to surgery was not associated with IMH closure, which was surprising given its positive association with MLD. 

### 4.2. Effect of Language 

Language barriers present a challenge in healthcare as they can lead to miscommunication, reduced patient satisfaction and decreased quality of healthcare delivery, ultimately impacting patient safety and overall outcomes [23,24]. Non-English- and Spanish-speaking groups had a larger mean MLD, which suggests there could be a delay in care for non-English speakers. Fear of stigma and discrimination have been cited as communication barriers [25] and may discourage patients from seeking medical care. 

### 4.3. Diabetes in the Hispanic Population and FTMH

Another possible contributing factor to larger MLD in certain populations is underlying comorbidities. Hispanic study eyes with diabetes were found to have significantly larger MLD and BD compared to Hispanic study eyes without diabetes. The Hispanic population has been reported to have an 80% higher rate of diabetes than non-Hispanic counterparts [26] and has been reported to experience diabetes-related complications at a greater rate [27,28,29]. One of the proposed reasons for this is decreased access to healthcare [26] and disadvantaged socioeconomic status [29]. Diabetic retinopathy has been associated with the formation of FTMH [30], and severity of diabetes within Hispanic patients may have contributed to the increased severity of IMH presentation observed in the current study. 

### 4.4. Visual Acuity Analysis

Preoperative VA has been reported to be a significant factor for predicting postoperative visual outcomes, as was observed in the current study [21,31]. The Hispanic ethnic group, and the Asian, Black, and Other racial groups all presented with worse preoperative VA, suggesting more severe presentation and increased risk of worse outcomes. 

Although there were significant differences in postoperative VA at the 12-month follow-up visits, true change in VA can be masked in phakic patients following PPV [32] due to accelerated cataract formation within the first 6 months postoperatively [32,33]. This suggests greater follow-up time and postoperative phakic status should be recorded in order to properly analyze postoperative VA. 

### 4.5. Age and Sex

Within the current study, the majority of patients were female, a sex distribution that is consistent with prior studies (68.7–76.7%) [4,34,35]. This preponderance of females was similarly identified among all ethnic and racial groups, which suggests that IMH development is more common among females regardless of ethnicity or race. Based on prior studies, the average age of patients presenting with IMH has been reported to be 62.6–68.6 years [34,35]. Consistent with this, the average age among patients within the current study was 66.3 years. Hispanic patients (64.0 years) were significantly younger than non-Hispanic patients (66.7 years), and significant differences in age were also observed between different racial groups (Asian = 61.8 years, Black = 63.7 years, White = 63.0 years, and Other = 67.6 years); these findings deserve further study and suggest that presenting age may vary across different ethnic and racial groups for the presentation of IMH.

It is important to consider the application of these findings in the clinical setting. Emphasis should be placed on broad education to all patient populations about the importance of seeking care when visual symptoms occur. Frequent communication with all patients throughout the surgery scheduling process should be attempted to encourage prompt treatment. Some populations were found to have a trend for decreased follow-up. Close monitoring to ensure adequate postoperative follow-up is encouraged. The intention of these suggestions is to assist in minimizing the disparities that were observed in this study. 

The strengths of the current study include a large sample size and novel evaluation of the Hispanic patient population. As a retrospective study, there are inherent limitations of this research. Other limitations include the lack of analysis of the surgical procedures studied in terms of gas or gauge type and surgical technique. Lack of complete follow-up and lack of refracted visual outcomes are additional limitations. Not accounting for cataract status at postoperative visits also limits the reliability of VA measurements. Analysis of insurance coverage would also provide further insight into barriers that could be contributing to health disparities observed in this study. Additional sensitivity analysis for follow-up beyond baseline differences between patients who underwent successful surgery was not performed. Repeatability of OCT measurements was not assessed, which would have strengthened the reliability of the MLD and BD measurements. 

## 5. Conclusions

Overall, care must be taken when evaluating the influence of race and ethnicity as these demographic characteristics are intertwined with socioeconomics, ancestry, culture, social determinants of health and various other factors which impact health outcomes. In summary, this retrospective analysis found that the Hispanic ethnic group and Black and Other racial groups had: (1) larger mean presenting MLD, (2) larger mean presenting BD, (3) worse VA at presentation, and (4) longer time to surgery. The Hispanic population was also found to have a larger proportion of eyes with large IMH. These findings highlight ethnic and racial variations in the presentation and surgical outcomes of IMH, and additional analyses are warranted to better understand these differences and approaches to optimizing outcomes for all patient subgroups. 

## Figures and Tables

**Table 1:** Patient demographics and clinical characteristics.

Demographic Data	
Number of Participants	488
Number of Eyes	515
OD: *n* (%)	252 (48.9)
OS: *n* (%)	263 (51.1)
Male: *n* (%)	145 (28.2)
Female: *n* (%)	370 (71.8)
Age: mean (SD)	66.3 (7.5)
Pseudophakic: *n* (%)	164 (31.8)
FE FTMH: *n* (%)	55 (10.7)
FE LMH: *n* (%)	23 (5.5)
**Population by Ethnicity**	***n* (%)**
Hispanic	79 (15.3)
Non-Hispanic	436 (84.7)
**Population by Race**	***n* (%)**
Asian	23 (4.5)
Black	79 (15.3)
White	365 (70.9)
Other	48 (9.3)
**Ethnicity**	**Mean Age (SD)**
Hispanic	64.0 (9.5)
Non-Hispanic	66.7 (7.0)
*p*-Value	0.016 *
**Race**	**Mean Age (SD)**
Asian	61.8 (7.5)
Black	63.7 (7.3)
White	63.0 (6.6)
Other	67.6 (10.4)
*p*-Value	<0.001 *
**Language**	***n* (%)**
English	475 (92.2)
Spanish	33 (6.4)
Other	7 (1.4)
**Diabetes**	***n* (%)**
Type I	2 (0.4)
Type II	78 (15.1)

FE = fellow eye; FTMH = full-thickness macular hole; LMH = lamellar macular hole. * *p*-value < 0.05.

**Table 2:** Mean time to surgery (days) categorized by ethnicity, race, preferred language, and diabetes status.

Covariate	Mean Time to Surgery (SD), days				*p*-Value
**Ethnicity**	*Hispanic*	*Non-Hispanic*			
	99.7 (245.8)	43.2 (68.4)			0.046 *
**Race**	*Asian*	*Black*	*White*	*Other*	
	30.6 (62.5)	64.2 (101.8)	39.5 (56.2)	135.9 (309.5)	<0.001 *
**Preferred Language**	*English*	*Non-English*			
	50.0 (115.2)	73.9 (129.0)			0.263
	*Spanish*	*Non-Spanish*			
	92.9 (139.2)	50.7 (114.5)			0.127
**Diabetes**	*Diabetic*	*Non-Diabetic*			
	48.3 (94.6)	52.5 (120.1)			0.723

* *p*-value < 0.05.

**Table 3:** Preoperative logMAR VA of the study eyes categorized by ethnicity, race, and IMH closure status.

Covariate	Mean Preoperative LogMAR Visual Acuity (SD)				*p*-Value
**Ethnicity**	*Hispanic*	*Non-Hispanic*			
	1.00 (0.48)	0.84 (0.40)			0.008 *
**Race**	*Asian*	*Black*	*White*	*Other*	
	1.01 (0.45)	0.95 (0.43)	0.81 (0.38)	1.10 (0.51)	<0.001 *
IMH Status **	*Closed*	*Open*			
	0.85 (0.41)	1.14 (0.45)			0.003 *

IMH = idiopathic macular hole; logMAR = logarithm of the minimum angle of resolution; VA = visual acuity. * *p*-value < 0.05. ** Preoperative visual acuity of successful (closed) and unsuccessful (open) surgical outcomes.

**Table 4:** Mean MLD and BD categorized by ethnicity, race, preferred language, and diabetes status.

Covariate	Mean Measurement (SD), μm				*p*-Value
**Sex**	*Female*	*Male*			
	*(n = 370)*	*(n = 145)*			
MLD	389.3 (200.5)	307.0 (175.7)			<0.001 *
BD	756.9 (349.2)	728.8 (358.3)			0.420
**Ethnicity**	*Hispanic* *(n = 79)*	*Non-Hispanic* *(n = 436)*			
MLD	437.9 (219.0)	353.2 (190.1)			0.002 *
BD	861.7 (417.2)	728.5 (334.9)			0.009 *
**Race**	*Asian* *(n = 23)*	*Black* *(n = 79)*	*White* *(n = 365)*	*Other* *(n = 365)*	
MLD	428.0 (208.9)	467.4 (220.1)	327.2 (172.9)	466.4 (227.3)	<0.001 *
BD	764.2 (300.1)	812.7 (352.2)	712.8 (331.7)	912.0 (456.1)	<0.001 *
**Preferred Language**	*English* *(n = 451)*	*Non-English* *(n = 37)*			
MLD	357.5 (190.6)	469.0 (242.9)			0.007 *
BD	742.1 (344.8)	830.5 (421.4)			0.204
	*Spanish* *(n = 30)*	*Non-Spanish* *(n = 458)*			
MLD	482.0 (224.5)	357.6 (193.1)			0.007 *
BD	873.2 (428.7)	742.2 (344.9)			0.121
**Diabetes**	*Diabetic* *(n = 78)*	*Non-Diabetic* *(n = 435)*			
MLD, Overall	359.3 (177.2)	367.4 (200.8)			0.713
MLD, Non-Hispanic	326.4 (169.2)	357.9 (193.5)			0.176
MLD, Hispanic	514.2 (128.2)	421.5 (232.6)			0.046 *
BD, Overall	747.4 (385.9)	749.3 (345.5)			0.968
BD, Non-Hispanic	684.5 (376.9)	736.4 (326.8)			0.296
BD, Hispanic	1044.1 (282.4)	822.4 (432.6)			0.024 *

BD = basal diameter; MLD = minimum linear diameter. * *p*-value < 0.05.

**Table 5:** Number of small, medium, and large IMH by MLD and BD categorized by ethnicity.

Size of IMH	Number of Study Eyes	
***Ethnicity***	*Hispanic (n = 79)*	*Non-Hispanic (n = 436)*
**MLD**		
Small	20	147
Medium	14	134
Large	45	155
*p*-value	<0.001 *	0.462
**BD**		
Small	21	153
Medium	17	152
Large	41	131
*p*-value	0.002 *	0.346

BD = basal diameter; IMH = idiopathic macular hole; MLD = minimum linear diameter. * *p*-value < 0.05.

**Table 6:** Number of small, medium, and large IMH by MLD and BD categorized by race.

Size of IMH	Number of Study Eyes			
***Race***	*Asian (n = 23)*	*Black (n = 79)*	*White (n = 365)*	*Other (n = 48)*
**MLD**				
Small	4	13	140	10
Medium	7	20	113	8
Large	12	46	112	30
*p*-value	0.119	<0.001 *	0.126	<0.001 *
**BD**				
Small	6	23	132	13
Medium	8	22	131	8
Large	9	34	102	27
*p*-value	0.738	0.186	0.092	0.002 *

BD = basal diameter; IMH = idiopathic macular hole; MLD = minimum linear diameter. * *p*-value < 0.05.

**Table 7:** Macular hole closure following surgery categorized by ethnicity, race, language, FE FTMH, MLD size, BD size, diabetes status, mean MLD, mean BD, and mean time to surgery.

Covariant	Macular Hole Closure		*p*-Value
**Ethnicity**			0.399
**Race**			0.418
**Language**			
*English* vs. *non-English*			0.718
*Spanish* vs. *non-Spanish*			0.470
**FE FTMH**			0.267
**MLD Size**			<0.001 *
**BD Size**			<0.001 *
**Diabetes**			0.759
**MLD**	*Open [mean (SD), μm]*	*Closed [mean (SD), μm]*	
	607.3 (188.6)	353.3 (189.4)	<0.001 *
**BD**	*Open [mean (SD), μm]*	*Closed [mean (SD), μm]*	
	1119.7 (327.7)	729.3 (342.1)	<0.001 *
**TTS**	*Open [mean (SD), days]*	*Closed [mean (SD), days]*	
	55.7 (83.4)	51.7 (118.0)	0.815

BD = basal diameter; FE FTMH = fellow eye full thickness macular hole; MLD = minimum linear diameter; TTS = time to surgery (days). * *p*-value < 0.05.

**Table 8:** Mean preoperative logMAR VA of the study eyes categorized by ethnicity, race, and IMH closure status.

Covariate	Mean Postoperative LogMAR Visual Acuity (SD)				*p*-Value
**Ethnicity**	*Hispanic*	*Non-Hispanic*			
POM1	0.77 (0.51)	0.68 (0.47)			0.144
POM6	0.64 (0.50)	0.52 (0.42)			0.115
POM12	0.62 (0.51)	0.42 (0.40)			0.026 *
**Race**	*Asian*	*Black*	*White*	*Other*	
POM1	0.80 (0.38)	0.75 (0.48)	0.66 (0.49)	0.768 (0.46)	0.210
POM6	0.55 (0.27)	0.57 (0.42)	0.53 (0.44)	0.557 (0.47)	0.922
POM12	0.46 (0.30)	0.52 (0.45)	0.40 (0.39)	0.69 (0.55)	0.005 *
IMH Status **	*Closed*	*Open*			
POM1	0.66 (0.46)	1.23 (0.51)			<0.001 *
POM6	0.51 (0.41)	1.29 (0.54)			<0.001 *
POM12	0.42 (0.38)	1.53 (0.41)			<0.001 *

IMH = idiopathic macular hole; logMAR = logarithm of the minimum angle of resolution; POM1 = postoperative month 1; POM6 = postoperative month 6; POM12 = postoperative month 12; VA = visual acuity. * *p*-value < 0.05. ** Preoperative visual acuity of successful (closed) and unsuccessful (open) surgical outcomes.

## Data Availability

The data presented in this study are available on request from the corresponding author. The data are not publicly available due to privacy reasons.
